# The Role of *VEGF* and *KDR* Polymorphisms in Moyamoya Disease and Collateral Revascularization

**DOI:** 10.1371/journal.pone.0047158

**Published:** 2012-10-12

**Authors:** Young Seok Park, Young Joo Jeon, Hyun Seok Kim, Kyu Young Chae, Seung-Hun Oh, In Bo Han, Hyun Sook Kim, Won-Chan Kim, Ok-Joon Kim, Tae Gon Kim, Joong-Uhn Choi, Dong-Seok Kim, Nam Keun Kim

**Affiliations:** 1 Department of Neurosurgery, CHA Bundang Medical Center, CHA University School of Medicine, Seongnam, South Korea; 2 Institute for Clinical Research, CHA Bundang Medical Center, CHA University School of Medicine, Seongnam, South Korea; 3 Department of Pediatrics, CHA Bundang Medical Center, CHA University School of Medicine, Seongnam, South Korea; 4 Department of Neurology, CHA Bundang Medical Center, CHA University School of Medicine, Seongnam, South Korea; 5 Department of Pediatric Neurosurgery, Severance Hospital, Brain Korea 21 Project for Medical Science, Brain Research Institute, Yonsei University College of Medicine, Seoul, Korea; University of Queensland, Australia

## Abstract

We conducted a case-control study to investigate whether vascular endothelial growth factor (*VEGF* −2578, −1154, −634, and 936) and kinase insert domain containing receptor (*KDR* −604, 1192, and 1719) polymorphisms are associated with moyamoya disease. Korean patients with moyamoya disease (n = 107, mean age, 20.9±15.9 years; 66.4% female) and 243 healthy control subjects (mean age, 23.0±16.1 years; 56.8% female) were included. The subjects were divided into pediatric and adult groups. Among the 64 surgical patients, we evaluated collateral vessel formation after 2 years and divided patients into good (collateral grade A) or poor (collateral grade B and C) groups. The frequencies and distributions of four *VEGF* (−2578, −1154, −634, and 936) and *KDR* (−604, 1192, and 1719) polymorphisms were assessed from patients with moyamoya disease and compared to the control group. No differences were observed in *VEGF* −2578, −1154, −634, and 936 or *KDR −*604, 1192, and 1719 polymorphisms between the control group and moyamoya disease group. However, we found the −634CC genotype occurred less frequently in the pediatric moyamoya group (*p* = 0.040) whereas the *KDR* −604C/1192A/1719T haplotype increased the risk of pediatric moyamoya (*p* = 0.024). Patients with the CC genotype of *VEGF* −634 had better collateral vessel formation after surgery. Our results suggest that the *VEGF* −634G allele is associated with pediatric moyamoya disease and poor collateral vessel formation.

## Introduction

Although many studies have investigated the etiology of moyamoya disease, unsatisfactory progress has been made. Moyamoya disease etiology may be idiopathic, environmental, or genetic. The search for genetic loci linked to moyamoya disease has uncovered associations with chromosomes 3, 6, 7, 8, and 17 and the HLA haplotype [1]–[8], but relevant genes have not been identified [9]. Recent linkage analyses from East Asian families with moyamoya disease demonstrated a linkage of 17q25.3 with the disease at a locus that is −1480 bp from the transcription site of the Raptor gene. However, an allele of this gene was not detected in samples from Caucasian patients with this disease [10].

Vascular endothelial growth factor (VEGF) is involved in vasculogenesis and vascular permeability in various intracranial lesions [11]. In ischemic disease, cerebral angiogenesis is caused by the release of VEGF [12], [13]. VEGF affects vasculogenesis, endothelial cell proliferation and migration, vascular permeability, and stromal degradation through the activation of proteolytic enzymes that are involved in angiogenesis [14], [15]. VEGF binds its receptor tyrosine kinases, VEGF receptor-1 and VEGF receptor-2 (also known as kinase insert domain containing receptor, or KDR) but KDR is the key receptor mediating angiogenesis [16] and is essential for endothelial cell survival and integrity [17].

Although excess VEGF in moyamoya disease has been demonstrated [11], [18] and the association is convincing, the specific role for *VEGF* remains unclear. Therefore, we studied the relationship of *VEGF* and *KDR* polymorphisms and moyamoya disease. VEGF is a major angiogenic factor and a prime regulator of endothelial cell proliferation [19]. The gene that encodes *VEGF* is located on chromosome 6 and is comprised of a 14-kb coding region with eight exons and seven introns [20]. VEGF is activated transcriptionally and posttranscriptionally by hypoxia in tumor necrosis and in various models of ischemia [21], [22]. Ischemia stimulates *VEGF* expression in the brain suggesting that it may be important for the vascular response to cerebral ischemia [23], [24], [25]. Several single nucleotide polymorphisms (SNPs) have been described in the *VEGF* gene (National Center for Biotechnology Information, Gene association no: NT 007592). The *VEGF* gene includes at least 4 relatively common polymorphisms that may influence *VEGF* expression: −2578C>A (rs699947), −1154G>A (rs1570360), −634G>C (rs2010963), and 936C>T (rs3025039) [26], [27], [28].

**Table 1:** Demographic characteristics between controls and moyamoya patients.

Characteristic	Control (n = 243)	Moyamoya(n = 107)	*P* *	Ischemicmoyamoya(n = 92)	Hemorrhagic moyamoya(n = 15)	*P* *
Number of subjects						
<18 years	102 (42.0)	56 (52.3)		54 (58.7)	2 (13.3)	
≥18 years	141 (58.0)	51 (47.7)		38 (41.3)	13 (86.7)	
Age (means±SD)						
<18 years	7.71±4.05	7.98±4.13	0.920	8.11±4.12	4.50±3.54	NA
≥18 years	36.72±10.05	34.98±11.29	0.248	34.63±11.45	36.00±11.21	0.689
Sex [male, n(%)]						
<18 years	54 (52.9)	22 (39.3)	0.100	21 (38.9)	1 (50.0)	1.000†
≥18 years	51 (21.0)	14 (27.5)	0.260	12 (31.6)	2 (15.4)	0.472†
Collateral vessel formation score (n = 64)						
0	–	2				
1	–	22				
2	–	40				

* *P* values were calculated using the Mann-Whitney test for continuous data and χ^2^-test for categorical data.

† Fisher's exact test. NA; not applicable.

Three of these polymorphisms are located in the promoter region at −2578, −1154, and −634 relative to the translation start site. The −2578A, −1154A, and −634G alleles are all associated with decreased *VEGF* expression [26], [27]. In addition to promoter region polymorphisms, the T allele of the common 936C>T polymorphism in the 3′-untranslated region is also associated with significantly decreased serum VEGF levels [28]. Recently, several SNPs of the VEGF gene have been associated with cancer risk and prognosis [29], as well as coronary arterial disease [30]. Moreover, *KDR* −604C, 1192A, and 1719A alleles of chromosome 4 were associated with decreased VEGF binding activity and coronary artery disease [31]. These results indicate the importance of VEGF-KDR signaling in human disease.

**Figure 1:**
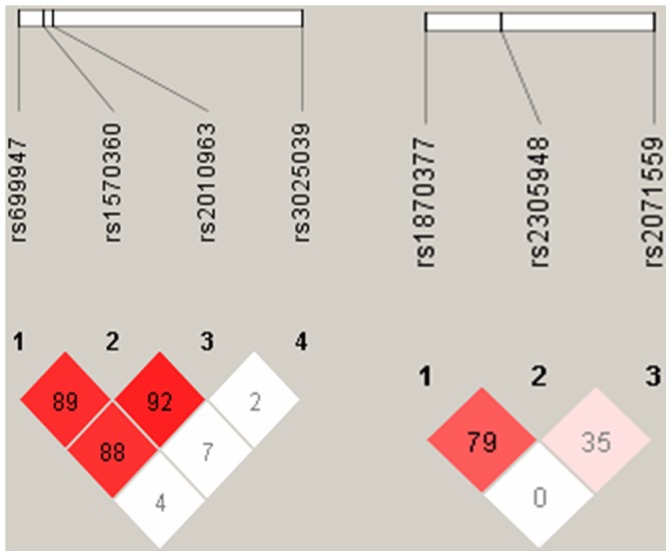
Linkage disequilibrium(LD) of VEGF and KDR polymorphisms. There was strong LD between −1154G>A (rs 1570460) and −634G>C (rs2010963) (D′ = 0.92), −2578C>A (rs 699947)and 1154G>A (rs1570360) (D′ = 0.89). There was strong LD between 1719T>A(rs1870377) and 1192G>A (rs2305948) (D′ = 0.79).

**Table 2:** The frequencies of the *VEGF* −2578C>A, −1154G>A, −634G>C, and 936C>T polymorphisms between control subjects and patients with moyamoya disease.

Characteristics	Controls (n = 243, %)	Moyamoya patients (n = 107, %)	AOR (95% CI)	*P* *
*VEGF* −2578C>A				
CC	128 (52.7)	62 (57.9)	1.000 (reference)	
CA	99 (40.7)	36 (33.6)	0.769 (0.470–1.258)	0.296
AA	16 (6.6)	9 (8.4)	1.188 (0.491–2.873)	0.702
Dominant (CC vs. CA+AA)			0.827 (0.520–1.315)	0.422
Recessive (CC+CA vs. AA)			1.296 (0.547–3.072)	0.556
*VEGF–*1154G>A				
GG	173 (71.2)	80 (74.8)	1.000 (reference)	
GA	62 (25.5)	25 (23.4)	0.873 (0.510–1.496)	0.622
AA	8 (3.3)	2 (1.9)	0.517 (0.106–2.519)	0.414
Dominant (GG vs. GA+AA)			0.834 (0.495–1.405)	0.495
Recessive (GG+GA vs. AA)			0.546 (0.113–2.653)	0.454
*VEGF* −634G>C				
GG	90 (37.0)	37 (34.6)	1.000 (reference)	
GC	103(42.4)	48 (44.9)	1.127 (0.666–1.906)	0.655
CC	50 (20.6)	22 (20.6)	1.083 (0.575–2.042)	0.804
Dominant (GG vs. GC+CC)			1.124 (0.695–1.817)	0.634
Recessive (GG+GC vs. CC)			1.024 (0.579–1.811)	0.934
*VEGF* 936C>T				
CC	172 (70.8)	67 (62.6)	1.000 (reference)	
CT	64 (26.3)	37 (34.6)	1.530 (0.926–2.529)	0.097
TT	7 (2.9)	3 (2.8)	1.230 (0.301–5.027)	0.773
Dominant (CC vs. CT+TT)			1.484 (0.912–2.416)	0.112
Recessive (CC+CT vs. TT)			1.079 (0.270–4.313)	0.915

* Adjusted by age and gender. AOR, adjusted odds ratio. CI, confidence interval.

To our knowledge, no previous study has evaluated both *KDR* and *VEGF* polymorphisms with moyamoya disease and collateral vessel formation after surgery. The aim of present study was to evaluate the frequencies of the *VEGF* −2578C>A, −1154G>A, −634G>C, and 936C>T and *KDR* −604T>C (rs2071559), 1192G>A (rs2305948), and 1719T>A (rs1870377) polymorphisms in Korean patients with moyamoya disease in an effort to determine the relationship of these polymorphisms with moyamoya disease.

**Table 3:** The frequencies of the *KDR* −604T>C, 1192G>A, and 1719T>A polymorphisms between control subjects and patients with moyamoya disease.

Characteristics	Controls (n = 243, %)	Moyamoya patients (n = 107, %)	AOR (95% CI)	*P* *
*KDR* −604T>C				
TT	113 (46.5)	47 (43.9)	1.000 (reference)	
TC	108 (44.4)	50 (46.7)	1.131 (0.698–1.833)	0.617
CC	22 (9.1)	10 (9.3)	1.102 (0.482–2.517)	0.818
Dominant (CC vs. CA+AA)			1.128 (0.710–1.792)	0.609
Recessive (CC+CA vs. AA)			1.039 (0.470–2.296)	0.925
*KDR* 1192G>A				
GG	186 (76.5)	80 (74.8)	1.000 (reference)	
GA	52 (21.4)	22 (20.6)	0.994 (0.563–1.754)	0.983
AA	5 (2.1)	5 (4.7)	2.140 (0.596–7.693)	0.244
Dominant (GG vs. GA+AA)			1.101 (0.646–1.875)	0.725
Recessive (GG+GA vs. AA)			2.139 (0.597–7.665)	0.243
*KDR* 1719T>A				
TT	46 (18.9)	26 (24.3)	1.000 (reference)	
TA	114 (46.9)	45 (42.1)	0.764 (0.417–1.401)	0.384
AA	83 (34.2)	36 (33.6)	0.799 (0.427–1.495)	0.482
Dominant (TT vs. TA+AA)			0.775 (0.446–1.346)	0.365
Recessive (TT+TA vs. AA)			0.997 (0.613–1.620)	0.989

* Adjusted by age and gender. AOR, adjusted odds ratio. CI, confidence interval.

## Methods

### Subjects

One hundred seven consecutive Korean patients with moyamoya disease [mean age, 20.9±15.9 years; 71 females (66.4%), 36 males (33.6%)] were recruited for this study. Moyamoya disease was defined as the presence of clinical ischemic or hemorrhagic symptoms in combination with vascular lesions in magnetic resonance imaging (MRI) or magnetic resonance angiography (MRA).

**Table 4:** The frequency of the *VEGF* polymorphisms according to age.

	Age <18 years				Age ≥18 years			
Characteristics	Controls (n = 102, %)	Moyamoya (n = 56, %)	AOR (95% CI)	*P* *	Controls (n = 141, %)	Moyamoya (n = 51, %)	AOR (95% CI)	*P* *
*VEGF* −2578C>A								
CC	57 (55.9)	29 (51.8)	1.000 (reference)		71 (50.4)	33 (64.7)	1.000 (reference)	
CA	42 (41.2)	22 (39.3)	1.100 (0.548–2.206)	0.789	57 (40.4)	14 (27.5)	0.539 (0.261–1.113)	0.095
AA	3 (2.9)	5 (8.9)	3.191 (0.696–14.631)	0.135	13 (9.2)	4 (7.8)	0.669 (0.200–2.242)	0.514
Dominant (CC vs. CA+AA)			1.246 (0.638–2.433)	0.519			0.543 (0.277–1.063)	0.075
Recessive (CC+CA vs. AA)			3.044 (0.686–13.499)	0.143			0.777 (0.239–2.525)	0.674
*VEGF* −1154G>A								
GG	77 (75.5)	44 (78.6)	1.000 (reference)		109 (77.3)	36 (70.6)	1.000 (reference)	
GA	22 (21.6)	9 (16.1)	1.614 (0.755–3.449)	0.217	30 (21.3)	13 (25.5)	0.391 (0.160–0.957)	0.040
AA	3 (2.9)	3 (5.4)	0.695 (0.067–7.200)	0.760	2 (1.4)	2 (3.9)	0.445 (0.049–4.029)	0.472
Dominant (GG vs. GA+AA)			1.522 (0.729–3.178)	0.264			0.395 (0.169–0.923)	0.032
Recessive (GG+GA vs. AA)			0.608 (0.061–6.044)	0.671			0.517 (0.058–4.589)	0.554
*VEGF* −634G>C								
GG	34 (33.3)	24 (42.9)	1.000 (reference)		56 (39.7)	13 (25.5)	1.000 (reference)	
GC	46 (45.1)	27 (48.2)	0.813 (0.398–1.664)	0.571	57 (40.4)	21 (41.2)	1.715 (0.771–3.816)	0.186
CC	22 (21.6)	5 (8.9)	0.301 (0.096–0.946)	0.040	28 (19.9)	17 (33.3)	2.698 (1.137–6.403)	0.024
Dominant (GG vs. GC+CC)			0.644 (0.236–1.273)	0.206			2.016 (0.979–4.152)	0.057
Recessive (GG+GC vs. CC)			0.334 (0.117–0.955)	0.041			2.087 (1.014–4.295)	0.046
*VEGF* 936C>T								
CC	76 (74.5)	38 (67.9)	1.000 (reference)		96 (68.1)	31 (60.8)	1.000 (reference)	
CT	24 (23.5)	18 (32.1)	1.580 (0.756–3.303)	0.224	41 (29.1)	17 (33.3)	1.322 (0.652–2.681)	0.438
TT	2 (2.0)	0 (0.0)	NA	NA	4 (2.8)	3 (5.9)	2.562 (0.525–12.506)	0.245
Dominant (CC vs. CT+TT)			1.405 (0.681–2.900)	0.357			1.378 (0.702–2.702)	0.351
Recessive (CC+CT vs. TT)							2.207 (0.472–10.328)	0.315

* Adjusted by age and gender. NA; not applicable. AOR, adjusted odds ratio. CI, confidence interval.

The control group was comprised of 243 healthy subjects [mean age, 23.0±16.1 years; 138 female (56.8%); 105 male (43.2%)] from the same geographic region as the moyamoya patients. These age- and sex-matched subjects were recruited from outpatient clinics at Severance Hospital (Seoul, Korea) and CHA Bundang Medical Center (Seongnam, Korea).

**Table 5:** The frequency of the *KDR* polymorphisms according to age.

		Age <18 years				Age ≥18 years			
Characteristics		Controls (n = 102, %)	Moyamoya (n = 56, %)	AOR (95% CI)	*P* *	Controls (n = 141, %)	Moyamoya (n = 51, %)	AOR (95% CI)	*P* *
*KDR* −604T>C									
CC		54 (52.9)	25 (44.6)	1.000 (reference)		59 (41.8)	22 (43.1)	1.000 (reference)	
CA		40 (39.2)	27 (48.2)	1.413(0.711–2.808)	0.324	68 (48.2)	23 (45.1)	0.929 (0.467–1.847)	0.833
AA		8 (7.8)	4 (7.1)	1.096(0.298–4.031)	0.890	14 (9.9)	6 (11.8)	1.141 (0.388–3.356)	0.811
Dominant (CC vs. CA+AA)				1.350(0.697–2.616)	0.374			0.951 (0.495–1.826)	0.879
Recessive (CC+CA vs. AA)				0.889(0.251–3.143)	0.855			1.198 (0.433–3.320)	0.728
*KDR* 1192G>A									
GG		20 (19.6)	16 (28.6)	1.000 (reference)		26 (18.4)	10 (19.6)	1.000 (reference)	
GA		47 (46.1)	22 (39.3)	0.572 (0.246–1.323)	0.193	67 (47.5)	23 (45.1)	1.082 (0.440–2.664)	0.864
AA		35 (34.2)	18 (32.1)	0.654 (0.232–1.572)	0.342	48 (34.0)	18 (35.3)	0.965 (0.382–2.435)	0.940
Dominant (GG vs. GA+AA)				0.611 (0.284–1.313)	0.207			1.003 (0.440–2.286)	0.994
Recessive (GG+GA vs. AA)				0.927 (0.460–1.869)	0.833			1.069 (0.543–2.103)	0.848
*KDR* 1719T>A									
TT	77 (75.5)		44 (78.6)	1.000 (reference)		109 (77.3)	36 (70.6)	1.000 (reference)	
TA	22 (21.6)		9 (16.1)	0.739 (0.310–1.762)	0.495	30 (21.3)	13 (25.5)	1.283 (0.602–2.734)	0.518
AA	3 (2.9)		3 (5.4)	1.848 (0.349–9.773)	0.470	2 (1.4)	2 (3.9)	3.003 (0.403–22.390)	0.283
Dominant (TT vs. TA+AA)				0.877 (0.398–1.934)	0.746			1.378 (0.667–2.846)	0.386
Recessive (TT+TA vs. AA)				2.026 (0.382–10.736)	0.407			2.675 (0.362–19.768)	0.335

* Adjusted by age and gender. NA; not applicable. AOR, adjusted odds ratio. CI, confidence interval.

Moyamoya disease has a bimodal pattern of incidence so we divided the patients into pediatric (<18 years) and adult (≥18 years) groups. We further divided the moyamoya patients into ischemic or hemorrhagic groups based on clinical and MRI findings. We performed indirect bypass surgery in 64 patients and direct superficial temporal artery to middle cerebral artery bypass plus encephalo-duro-arterio-myo-synangiosis (STA-MCA plus EDAMS) in one patient. We graded newly developed collateral vessels according to the method of Matsushima et al. [32]. Briefly, Grade A represented synangiosis-induced filling of greater than two-thirds of MCA circulation, Grade B represented between one-third and two-thirds, and Grade C represented less than one-third. We further divided the 64 indirect bypass surgical patients by collateral vessel formation after 2 years into good (collateral grade A) and poor (collateral grade B, C) using MRA. Table 1 shows the demographic characteristics of the moyamoya patients and control subjects.

**Table 6:** Haplotype frequencies of the *VEGF a*nd *KDR* polymorphisms according to age.

	Age <18 years				Age ≥18 years			
Characteristics	Controls (n = 102, %)	Moyamoya (n = 56, %)	OR (95% CI)	*P* *	Controls (n = 141, %)	Moyamoya (n = 51, %)	OR (95% CI)	*P* *
*VEGF* −2578/−1154/−634/936								
CGCC	0.3502	0.2331	0.566 (0.335–0.957)	0.041	0.3251	0.4350	1.594 (1.001–2.540)	0.053
CGGC	0.2894	0.3561	1.365 (0.836–2.231)	0.254	0.2360	0.2047	0.842 (0.484–1.465)	0.584
AAGC	0.1234	0.1361	1.107 (0.557–2.199)	0.860	0.1471	0.0406	0.250 (0.087–0.718)	0.006
CGCT	0.0910	0.0578	0.551 (0.213–1.423)	0.277	0.0708	0.0741	0.976 (0.400–2.382)	1.000
AGGC	0.0840	0.0648	0.733 (0.295–1.826)	0.658	0.1085	0.0587	0.511 (0.207–1.265)	0.171
CGGT	0.0233	0.0372	1.474 (0.388–5.606)	0.726	0.0656	0.0620	0.926 (0.357–2.403)	1.000
AGGT	0.0152	0.0532	3.792 (0.930–15.470)	0.073	0.0189	0.0310	1.696 (0.398–7.231)	0.440
CAGC	0.0109	0.0221	1.836 (0.255–13.220)	0.617	NA	NA	NA	NA
AAGT	NA	NA	NA	NA	0.0149	0.0368	2.866 (0.703–11.680)	0.216
*KDR* −604/1192/1719								
TGA	0.4141	0.3786	0.857 (0.534–1.376)	0.550	0.3624	0.3736	1.048 (0.655–1.675)	0.845
TGT	0.2187	0.2688	1.293 (0.758–2.204)	0.407	0.2551	0.2337	0.897 (0.528–1.525)	0.689
CGA	0.1325	0.1393	1.093 (0.561–2.128)	0.864	0.1990	0.1919	0.984 (0.557–1.740)	0.957
CGT	0.0974	0.0794	0.804 (0.353–1.831)	0.687	0.0630	0.0341	0.444 (0.128–1.542)	0.190
TAT	0.0773	0.0402	0.549 (0.196–1.541)	0.346	0.0421	0.0495	1.160 (0.398–3.378)	0.786
CAT	0.0330	0.0938	3.065 (1.153–8.148)	0.024	0.0618	0.1042	1.884 (0.851–4.173)	0.113

* *P-values* after 10,000 permutation test. NA; not applicable. OR, odds ratio. CI, confidence interval.

All participants gave informed written consent prior to enrollment in the study. The institutional review boards of Severance Hospital (4-2008-0308) and CHA Bundang Medical Center (PBC09-103) approved this study.

**Table 7:** Comparison of the *VEGF* and *KDR* genotype frequencies according to collateral vessel formation score.

Characteristics	Collateral grade A (n = 40)	Collateral grade B and C (n = 24)	AOR (95% CI)	*P* *
*VEGF* −2578C>A				
CC	20 (50.0)	13 (54.2)	1.000 (reference)	
CA	18 (45.0)	7 (29.2)	0.615 (0.200–1.893)	0.397
AA	2 (5.0)	4 (16.7)	3.060 (0.473–19.816)	0.241
Dominant (CC vs. CA+AA)			0.867 (0.313–2.408)	0.785
Recessive (CC+CA vs. AA)			3.757 (0.625–22.590)	0.148
*VEGF* −1154G>A				
GG	27 (67.5)	17 (70.8)	1.000 (reference)	
GA	12 (30.0)	6 (25.0)	0.841 (0.253–2.796)	0.777
AA	1 (2.5)	1 (4.2)	1.475 (0.082–26.410)	0.792
Dominant (GG vs. GA+AA)			0.926 (0.297–2.886)	0.895
Recessive (GG+GA vs. AA)			1.565 (0.090–27.244)	0.759
*VEGF* −634G>C				
GG	11 (27.5)	13 (54.2)	1.000 (reference)	
GC	22 (55.0)	7 (29.2)	0.252 (0.076–0.837)	0.024
CC	7 (17.5)	4 (16.7)	0.284 (0.045–1.780)	0.179
Dominant (GG vs. GC+CC)			0.286 (0.095–0.859)	0.026
Recessive (GG+GC vs. CC)			0.857 (0.215–3.412)	0.827
*VEGF* 936C>T				
CC	27 (67.5)	16 (66.7)	1.000 (reference)	
CT	13 (32.5)	8 (33.3)	1.069 (0.357–3.206)	0.905
TT	0 (0.0)	0 (0.0)	NA	NA
Dominant (CC vs. CT+TT)			1.069 (0.357–3.206)	0.905
Recessive (CC+CT vs. TT)			NA	NA
*KDR* −604T>C				
TT	17 (42.5)	11 (45.8)	1.000 (reference)	
TC	19 (47.5)	12 (50.0)	0.950 (0.325–2.778)	0.926
CC	4 (10.0)	1 (4.2)	0.370 (0.035–3.962)	0.411
Dominant (TT vs. TC+CC)			0.840 (0.298–2.367)	0.741
Recessive (TT+TC vs. CC)			0.346 (0.035–3.409)	0.363
*KDR* 1192G>A				
GG	30 (75.0)	16 (66.7)	1.000 (reference)	
GA	8 (20.0)	7 (29.2)	1.381 (0.401–4.758)	0.609
AA	2 (5.0)	1 (4.2)	0.843 (0.063–11.263)	0.897
Dominant (GG vs. GA+AA)			1.355 (0.420–4.375)	0.612
Recessive (GG+GA vs. AA)			0.664 (0.053–8.390)	0.752
*KDR* 1719T>A				
TT	10 (25.0)	7 (29.2)	1.000 (reference)	
TA	18 (45.0)	9 (37.5)	0.786 (0.219–2.824)	0.712
AA	12 (30.0)	8 (33.3)	0.886 (0.227–3.462)	0.861
Dominant (TT vs. TA+AA)			0.825 (0.259–2.626)	0.745
Recessive (TT+TA vs. AA)			1.195 (0.398–3.593)	0.751

* Adjusted by age and gender. NA; not applicable. AOR, adjusted odds ratio. CI, confidence interval.

### VEGF Genotyping

We investigated four relevant single nucleotide polymorphism (SNP) candidates in the *VEGF* gene. We used the G-DEX blood extraction kit (iNtRON Biotechnology, Inc., Seongnam, South Korea) according to the manufacturer’s instructions for DNA extraction. We obtained all SNP sequences from the HapMap database (www.hapmap.org) [33]. The *VEGF* -2578C>A, -1154G>A, -634G>C, and 936C>T polymorphisms were analyzed by polymerase chain reaction–restriction fragment length polymorphism (PCR-RFLP) method.

We used following primers: *VEGF* −2578C>A polymorphism, forward 5′-GGA TGG GGC TGA CTA GGT AAG-3′ and reverse 5′-AGC CCC CTT TTC CTC CAA C-3′, that amplifies a 308 bp (C allele) or 326 bp (A allele) product; *VEGF* −1154G>A polymorphism, forward 5′-CGC GTG TCT CTG GAC AGA GTT TCC-3′ and reverse 5′-CGG GGA CAG GCG AGC TTC AG-3′, that amplifies a 173 bp product; *VEGF* −634G>C polymorphism, forward 5′-CAG GTC ACT CAC TTT GCC CCG GTC-3′ and reverse 5′-GCT TGC CAT TCC CCA CTT GAA TCG-3′, that amplifies a 204 bp product; and *VEGF* 936C>T polymorphism, forward 5′-AAG GAA GAG GAG ACT CTG CGC AGA GC-3′ and reverse 5′-TAA ATG TAT GTA TGT GGG TGG GTG TGT CTA CAG G-3′, that amplifies a 208 bp fragment.

The *VEGF* −2578C>A and −634G>C polymorphisms were identified by digesting the PCR product with the restriction endonuclease *Ava*II (New England Biolabs, Beverly, MA, USA). The *VEGF* −1154G>A polymorphism was identified by digesting the PCR product with the restriction endonuclease *Mnl*I (New England Biolabs). The *VEGF* 936C>T polymorphism was identified by digesting the PCR product with the restriction endonuclease *Nla*III (New England Biolabs). All restriction digests were performed at 37°C for 16 h.

### KDR Genotyping

We identified 3 well-known SNPs in the *KDR* gene, including one in the promoter region (−604) and two in the coding region (1192 and 1719). All SNP sequences were obtained from the HapMap database (http://www.hapmap.org) [33]. We used previously described primers and PCR-RFLP conditions for *KDR* polymorphism analyses [31]. For SNP −604, two DNA fragments (174 bp and 116 bp) were observed for the C allele and one band (290 bp) was produced for the T allele. For the 1192 polymorphism, two DNA fragments (30 bp and 232 bp) indicated the A allele and one band (252 bp) was produced for the G allele. For SNP 1719, two DNA fragments (191 bp and 213 bp) were observed for the A allele and one band (404 bp) was produced for the T allele. The genotyping reproducibility was confirmed by bi-directional sequencing of 400 randomly selected samples.

### Measurement of Plasma Total Homocysteine (tHcy), Folic Acid (FA), and Vitamin B12 (VB12)

Blood was collected from moyamoya patients into a tube containing anticoagulant 12 hours after a meal. The tube was centrifuged for 15 min at 1000 × *g*, and the plasma was separated. The concentration of tHcy (n = 37, 10.18±2.81 µmol/L) in the plasma was measured by fluorescent polarizing immunoassay (FPIA) with IMx (Abbott Laboratories, Chicago, IL, USA). The plasma concentration of FA (n = 30, 10.25±3.50 ng/ml) and VB12 (n = 35, 848.06±319.22 pg/ml) was determined using a radioassay kit (ACS 180; Bayer, Tarrytown, NY, USA).

### Measurement of Whole Blood Nitric Oxide (NO)

Blood was collected from moyamoya patients into a tube containing anticoagulant 12 hours after a meal. NO production was evaluated by determining the circulating levels of nitrosyl-hemoglobin complexes. In the present study, the paramagnetic properties of nitrosyl-heme adducts were used to detect whole blood NO-hemoglobin derivatives (n = 33, 5.30±6.93 arbitrary unit [AU]) by electron paramagnetic resonance (EPR) spectroscopy.

### Statistical Analyses

To analyze the demographic characteristics of moyamoya disease, we used the Mann–Whitney and chi-square (χ^2^) tests for continuous and categorical data, respectively. The associations among pediatric and adult patients were estimated by computing the odds ratios (ORs) and 95% confidence intervals (CIs) using Fisher’s exact test. The adjusted odds ratios (AORs) for *VEGF* and *KDR* polymorphisms were calculated using multiple logistic regression analyses using gender and age. The genotype distribution of each polymorphism was expected under Hardy-Weinberg equilibrium. Statistical analyses were performed using GraphPad Prism 4.0 (GraphPad Software, Inc., San Diego, CA, USA) and StatsDirect software (version 2.4.4; StatsDirect Ltd., Altrincham, UK). Haplotype analyses were performed using HAPSTAT (version 3.0; University of North Carolina, Chapel Hill, NC, USA) and Haploview 4.2 (Broad Institute, Cambridge, MA, USA). StatsDirect Statistical Software (Version 2.4.4; StatsDirect Ltd, Altrincham, UK) was used to calculate the adjusted OR and 95% CI. The linkage disequilibrium between loci was measured using the absolute value of Lewontin’s D [34].

## Results

Figure 1 shows the linkage disequilibrium of *VEGF* and *KDR* polymorphisms from the present study. A comparison of genotype frequencies between moyamoya patients and control subjects of the *VEGF* −2578C>A, −1154G>A, −634G>C, and 936C>T polymorphisms and the *KDR* −604T>C, 1192G>A, and 1719T>A polymorphisms is shown in Table 2. There were no statistically significant differences between moyamoya patients and controls in any of the polymorphisms evaluated (Tables 2 and 3).

In subgroup analyses (Tables 4 and 5), the CC genotype of the *VEGF* −634 was less frequent in pediatric moyamoya disease (*p* = 0.040; CC vs. GG) and comparison with the GG+GC genotype was also significantly different in pediatric moyamoya patients. In the adult subgroup, the *VEGF* −634CC genotype was more frequent in moyamoya disease (*p* = 0.024; CC vs. GG). The frequencies of the *KDR* polymorphisms in both the pediatric and adult subgroups were not significantly different.

We conducted haplotype analyses of the *VEGF* and *KDR* polymorphisms (Table 6). The C-G-C-C haplotype (*VEGF* −2578/−1154/−634/936) in pediatric moyamoya patients was significantly different. In addition, the C-A-T haplotype (*KDR* −604/1192/1719) increased the risk of pediatric moyamoya. In adult moyamoya, the A-A-G-C haplotype (*VEGF* −2578/−1154/−634) was significantly different whereas other haplotypes were not. We have estimated and provided several haplotype frequencies in Table 6.

We performed indirect bypass surgery in 64 patients. The genotypes containing the *VEGF* −634C allele had better collateral vessel formation after surgery whereas −634GG was associated with poor collateral grade (Table 7). We also investigated the haplotype frequency differences related to collateral grades. However, there were no statistical differences (Table S1). Our results potentially implicate the *VEGF* −634G>C polymorphism in the development of collateral vessel formation in moyamoya disease. The other *VEGF* and *KDR* polymorphisms we studied did not exhibit statistically significant differences in collateral vessel formation.

To assess the clinical significance of the *VEGF* and *KDR* polymorphisms, we surveyed the association between the studied polymorphisms and various vascular risk factors (tHcy, FA, VB12, and NO). Blood tHcy, FA, VB12, and NO are essential for vascular homeostasis regulation [35]. There were no significant distinctions according to *VEGF* polymorphisms but the *KDR* −604C (*p* = 0.017) and 1192A (*p* = 0.032) alleles were linked to decreased NO levels (Table S2). The association of the *KDR* −604 and 1192 polymorphisms with NO levels suggests that *KDR* haplotypes containing −604C or 1192A may adversely affect vascular homeostasis.

## Discussion

In this study, we found *VEGF* or *KDR* polymorphisms influence moyamoya disease in subgroup analyses as well as the formation of revascularization after bypass surgery. VEGF is involved in vasculogenesis in different intracranial lesions [11], is an endothelial cell mitogen that induces transient vascular leakage, and a potent angiogenic factor [36]. *VEGF* promotes angiogenesis in cerebral ischemia [11], [37] and causes pathologic vessel formation [12].

Moyamoya disease is characterized by the angiographic findings of arterial stenosis and occlusion of the circle of Willis [38]. It can lead to transient ischemic attacks or a cerebral infarction pattern in juveniles [39] and a hemorrhagic stroke pattern in adults [40], [41]. Takekawa et al. [18] reported increased VEGF expression in autopsy specimens from adults with moyamoya disease and Sakamoto et al. reported that the total meningeal cellularity and VEGF expression in the moyamoya dura was significantly higher in moyamoya patients compared to controls [11]. Although increased VEGF concentrations have been demonstrated in moyamoya disease [11], [18], the specific role of *VEGF* in moyamoya genetics remains unclear. We therefore reasoned that mutations and genetic polymorphisms of the *VEGF* gene may cause cerebral ischemia in moyamoya disease.

Vascular endothelial growth factor A (VEGF-A) is a disulfide-bonded dimeric glycoprotein that is a member of a protein family that includes VEGF-B, VEGF-C, VEGF-D, and placental growth factor (PGF). The gene that encodes *VEGF-A* is located on human chromosome 6 and comprises a 14-kb coding region with eight exons. *VEGF* cellular signaling activity depends on specific membrane receptors. The receptors include fms like tyrosine kinase-1 (Flt-1 or VEGFR-1), KDR (also known as VEGFR-2) [42], [43], and Flt-4 (also known as VEGFR-3) [44], [45]. VEGF acts on endothelial cells particularly through Flt-1 and KDR [46], [47]. Epidermal growth factor (EGF) arising from hypoxia stimulates Flt-1 expression and inhibits KDR expression [48].

VEGF binding to KDR activates multiple signaling cascades that affect angiogenesis as well as endothelial cell survival, proliferation, and migration. Recently, several SNPs in the *VEGF* gene have been linked to cancer risk and prognosis [29] and coronary arterial disease [30], indicating the importance of the VEGF-KDR signaling pathway in human disease. Oh et al. reported the SNP 1719T allele conferred ischemic stroke risk in a dose dependent manner [49].

Revascularization after bypass surgery and the formation of new pial vessels may play a role in moyamoya disease. Several studies have suggested that endogenous VEGF production mediates compensatory revascularization during various physiological and pathological processes [37], [50], [51], [52].

In addition, as suggested by the data in our study, *VEGF* −634 G>C may be a possible prognostic biomarker after bypass surgery. Taken together, we can speculate that *VEGF* polymorphisms influence moyamoya disease as well as the formation of synangiosis-induced collateral vessel after bypass surgery. Although revascularization with pial synangiosis helps ameliorate ischemic symptoms, some patients progress to cerebral infarction or hemorrhage, even after surgery. This likely reflects the degree of synangiosis and is dependent on the genetic characteristics of the patient. Therefore, *VEGF* or *KDR* polymorphisms can be used as prognostic factors after revascularization surgery.

There are some limitations in correlating *VEGF* or *KDR* polymorphisms with our clinical findings. Our data should be interpreted with caution because of the relatively small sample size. Patients require long-term follow up to assess clinical outcomes and variation in the clinical characteristics of moyamoya disease makes it difficult to identify specific genes that are associated with the disease. In addition, moyamoya disease is characterized by genetic heterogeneity and complex interactions between genes and other factors. Although there are limitations with sample size and long-term follow-up clinical findings, it is important to determine the relationship of *VEGF* or *KDR* polymorphisms with moyamoya disease and collateral vessel formation after surgery.

In summary, no differences in *VEGF −*2578, −1154, −634, and 936 or *KDR* −604, 1192, and 1719 polymorphisms were observed between total moyamoya disease patients and control subjects. However, in subgroup analyses, we found that the CC genotype in *VEGF −*634 occurred less frequently in pediatric patients (*p* = 0.040) and occurred more often in adult moyamoya patients (*p* = 0.024). The genotypes including the *VEGF* −634C allele had better collateral vessel formation after surgery. In addition, the C-G-C-C (*VEGF* −2578/−1154/−634/936) haplotype and the C-A-T (*KDR* -604/1192/1719) haplotype in pediatric patients, as well as the A-A-G-C (*VEGF* −2578/−1154/−634/936) in adult moyamoya patients, had significant differences. Therefore, these results suggest that *VEGF* or *KDR* polymorphisms influence moyamoya disease as well as the formation of synangiosis-induced collateral vessel after bypass surgery.

## Supporting Information

Table S1 **Haplotype analyses of** ***VEGF*** **and** ***KDR*** **polymorphisms according to collateral score.** *P-values after 10,000 permutation test.
(DOC)

Table S2 **Association of** ***VEGF*** **and** ***KDR*** **polymorphisms with vascular risk factors.** *P-values between major and minor alleles of each polymorphism analyzed by Mann-Whitney test. AU; arbitrary units.
(DOC)
