# Supplementary material for: The Role of VEGF and KDR Polymorphisms in Moyamoya Disease and Collateral Revascularization
Source: PLoS One. 2012 Oct 12;7(10):e47158. doi: 10.1371/journal.pone.0047158 (PMC3470587; doi:10.1371/journal.pone.0047158)
Supplement: Table S1 — Haplotype analyses of VEGF and KDR polymorphisms according to collateral score. *P-values after 10,000 permutation test. (DOC) [file pone.0047158.s001.doc]

| **Table S1. Haplotype analyses of *VEGF* and *KDR* polymorphisms according to collateral score.** | | | | |
| --- | --- | --- | --- | --- |
| Haplotypes | Collateral score A (n=40) | Collateral score B or C (n=24) | OR (95% CI) | *P** |
| *VEGF* -2578/-1154/-634/936 | |  |  |  |
| CGCC | 0.3986 | 0.2840 | 0.618 (0.287 - 1.330) | 0.256 |
| CGGC | 0.2142 | 0.2744 | 1.376 (0.599 - 3.164) | 0.520 |
| AAGC | 0.1202 | 0.1000 | 0.814 (0.261 - 2.542) | 0.785 |
| AGGC | 0.0766 | 0.1214 | 1.762 (0.534 - 5.812) | 0.364 |
| CGGT | 0.0329 | 0.1042 | 2.985 (0.680 - 13.105) | 0.150 |
| CGCT | 0.0514 | 0.0000 | 0.175 (0.009 - 3.330) | 0.296 |
| AGGT | 0.0513 | 0.0208 | 0.404 (0.044 - 3.729) | 0.650 |
| AAGT | 0.0270 | 0.0417 | 1.696 (0.231 - 12.455) | 0.630 |
| CAGC | 0.0279 | 0.0250 | 0.830 (0.073 - 9.408) | 1.000 |
| AGCC | 0.0000 | 0.0285 | 5.084 (0.203 - 127.431) | 0.375 |
| *KDR* -604/1192/1719 | |  |  |  |
| TGA | 0.3723 | 0.4022 | 1.092 (0.524 - 2.277) | 0.852 |
| TGT | 0.2756 | 0.2329 | 0.784 (0.341 - 1.803) | 0.678 |
| CGA | 0.1527 | 0.0925 | 0.515 (0.156 - 1.700) | 0.408 |
| CAT | 0.1354 | 0.0883 | 0.570 (0.171 - 1.904) | 0.410 |
| CGT | 0.0494 | 0.0848 | 1.727 (0.411 - 7.255) | 0.472 |
| TAT | 0.0146 | 0.0732 | 7.182 (0.778 - 66.301) | 0.065 |
| CAA | 0.0000 | 0.0261 | 5.084 (0.203 - 127.431) | 0.375 |
| **P-values* after 10,000 permutation test. | | | | |
