# Supplementary material for: The Role of VEGF and KDR Polymorphisms in Moyamoya Disease and Collateral Revascularization
Source: PLoS One. 2012 Oct 12;7(10):e47158. doi: 10.1371/journal.pone.0047158 (PMC3470587; doi:10.1371/journal.pone.0047158)
Supplement: Table S2 — Association of VEGF and KDR polymorphisms with vascular risk factors. *P-values between major and minor alleles of each polymorphism analyzed by Mann-Whitney test. AU; arbitrary units. (DOC) [file pone.0047158.s002.doc]

| **Table S2. Association of *VEGF* and *KDR* polymorphisms with vascular risk factors.** | | | | | | | | |
| --- | --- | --- | --- | --- | --- | --- | --- | --- |
| Characteristics | Homocysteine  (μmol/L) | *P** | Folic acid  (ng/ml) | *P** | Vitamin B12  (pg/ml) | *P** | Nitric oxide  (AU) | *P** |
| *VEGF* -2578C | 10.37±2.81 | 0.319 | 10.72±3.69 | 0.107 | 847.53±307.90 | 0.960 | 5.81±7.20 | 0.116 |
| *VEGF* -2578A | 9.55±2.69 |  | 8.83±2.21 |  | 850.00±359.55 |  | 3.00±4.75 |  |
| *VEGF* -1154G | 10.22±2.79 | 0.882 | 10.53±3.52 | 0.071 | 848.45±299.06 | 0.564 | 5.55±7.10 | 0.473 |
| *VEGF* -1154A | 9.88±2.94 |  | 8.13±2.24 |  | 844.57±480.33 |  | 3.23±4.41 |  |
| *VEGF* -634G | 9.88±2.83 | 0.311 | 10.04±3.40 | 0.524 | 864.59±326.11 | 0.516 | 5.48±7.96 | 0.456 |
| *VEGF* -634C | 10.61±2.71 |  | 10.54±3.61 |  | 824.69±307.55 |  | 5.06±5.19 |  |
| *VEGF* 936C | 10.24±2.79 | 0.824 | 10.48±3.64 | 0.490 | 843.53±295.11 | 0.847 | 5.39±6.39 | 0.463 |
| *VEGF* 936T | 9.95±2.87 |  | 9.48±2.82 |  | 864.67±398.33 |  | 4.93±8.89 |  |
| *KDR* -604T | 10.32±2.62 | 0.341 | 10.33±3.56 | 0.994 | 829.96±295.39 | 0.711 | 5.90±6.35 | 0.017 |
| *KDR* -604C | 9.86±3.18 |  | 10.05±3.34 |  | 893.30±369.67 |  | 3.93±7.96 |  |
| *KDR* 1192G | 10.32±2.68 | 0.127 | 9.88±3.36 | 0.058 | 840.09±324.87 | 0.354 | 6.18±7.43 | 0.032 |
| *KDR* 1192A | 9.56±3.29 |  | 12.07±3.59 |  | 890.82±280.01 |  | 2.03±2.22 |  |
| *KDR* 1719T | 10.21±2.86 | 0.884 | 10.58±3.13 | 0.347 | 809.79±272.77 | 0.561 | 5.32±7.93 | 0.412 |
| *KDR* 1719A | 10.16±2.75 |  | 9.96±3.76 |  | 884.20±353.64 |  | 5.28±5.37 |  |
| * *P-values* between major and minor alleles of each polymorphism analyzed by Mann-Whitney test. AU; arbitrary unit. | | | | | | | | |
